# Prevalence of Tinnitus in an Aging Population and Its Relation to Age and Hearing Loss

**DOI:** 10.1177/0194599820957296

**Published:** 2020-09-29

**Authors:** Berthe C. Oosterloo, Pauline H. Croll, Robert J. Baatenburg de Jong, M. Kamran Ikram, André Goedegebure

**Affiliations:** 1 Department of Otorhinolaryngology–Head and Neck Surgery, Erasmus University Medical Center, Rotterdam, the Netherlands; 2 Department of Epidemiology, Erasmus University Medical Center, Rotterdam, the Netherlands; 3 Department of Radiology and Nuclear Medicine, Erasmus University Medical Center, Rotterdam, the Netherlands; 4 Department of Neurology, Erasmus University Medical Center, Rotterdam, the Netherlands

**Keywords:** tinnitus, hearing loss, age related, epidemiology

## Abstract

**Objectives:**

Tinnitus is a common hearing-related disorder, which may have a large impact on daily life. With aging populations worldwide, it is important to gain insight in the occurrence of tinnitus at older ages and to understand its relationship with age-related hearing loss. We investigated the prevalence of tinnitus among a general aging population, across age strata and hearing status.

**Study Design:**

Cross-sectional.

**Setting:**

The population-based Rotterdam Study.

**Methods:**

A total of 6098 participants underwent tinnitus assessment, and 4805 had additional hearing assessment. We determined tinnitus prevalence per 5-year age groups. Hearing impairment was defined as ≥25–dB HL worse ear pure tone average (0.5, 1, 2, 4 kHz). We investigated with multivariable logistic regression the association between hearing impairment and tinnitus. Tinnitus handicap was assessed in 663 participants with daily tinnitus via the Tinnitus Handicap Inventory–screening version (THI-s).

**Results:**

Tinnitus was prevalent in 21.4% (n = 1304). Prevalent tinnitus was evenly distributed over 5-year age groups. Participants with hearing impairment were more likely to have tinnitus (odds ratio, 2.27; 95% CI, 1.92-2.69) as compared with those without hearing impairment. The median THI-s score was 4 (interquartile range, 0-10), indicating a slight handicap, and 14.6% of the participants reported a moderate or severe handicap (THI-s ≥16).

**Conclusions:**

In a general elderly population, 1 in 5 persons has tinnitus. Of those with tinnitus, for 1 per 10 persons, the presence of tinnitus interfered with daily life. Participants with hearing impairment were twice as likely to have tinnitus. Despite the age-dependent occurrence of hearing impairment, no such age dependency was found for tinnitus.

Tinnitus is a common disorder in the adult population.^1^ Tinnitus is defined as a sound that is heard in the absence of an objective external sound source. For some, tinnitus is not bothersome at all, whereas others might experience it as very disturbing and warranting health care.^2,3^ In spite of the clear definition of subjective tinnitus as a phenomenon in literature, there is no consensus about when tinnitus becomes pathologic. The lack of a gold standard for pathologic tinnitus leads to a variety in reported prevalence. In several studies based on populations aged ≥18 years, tinnitus prevalence ranges from 9% up to 35%.^1^

Various risk factors for tinnitus have been reported: otologic, audiologic, personal, socioeconomic, and disease related.^4^ It is generally accepted that hearing impairment is one of the leading risk factors associated with tinnitus.^5,6^ As the worldwide population is aging,^7^ the prevalence of age-related hearing impairment is increasing accordingly.^8,9^ As such, it can be expected that tinnitus prevalence will increase as well.^10^ However, limited data are available on the age dependency of tinnitus in a population of older adults. Most studies that investigated tinnitus and age dependency did so in middle-aged populations.^1^ Studies that investigated older populations reported prevalence numbers of 8.2% up to 30.3%, with several citing age dependency^11-15^ and others not.^16-22^ There is still a lack of understanding about the prevalence and age dependency of tinnitus in the general aging population and its association with age-related hearing loss.

Therefore, in this study, we aimed to determine (1) the prevalence of tinnitus in an aging population-based sample; (2) its age distribution and association with sex and highest achieved education, taking into account the potential underlying association with hearing impairment; and (3) the handicap associated with prevalent tinnitus.

## Methods

### Setting and Study Population

This cross-sectional study was embedded in the Rotterdam Study, a prospective population-based cohort study. The Rotterdam Study was initiated in 1989, and it investigates determinants and consequences of aging. Details of the study have been described elsewhere.^23^ The entire study population consists of 14,926 individuals aged ≥45 years living in the well-defined Ommoord district in the city of Rotterdam, the Netherlands.^23^ All participants were invited to undergo extensive examinations in the dedicated research center at study entry and subsequently every 3 to 4 years. In total, almost 80% of the inhabitants aged ≥50 years who were invited to participate in the study between February 2011 and December 2016 were tested, including audiometry. Participation rates did not significantly vary among age groups.

Tinnitus and hearing assessment were introduced into the core study protocol in 2011. Of the 6168 eligible participants, 6098 had complete case information and were included in the current study: these patients underwent home interview regarding the presence or absence of tinnitus, and 663 of them filled out the Tinnitus Handicap Inventory–screening version (THI-s) between 2011 and 2016. Of the participants with information on tinnitus status, 4805 underwent hearing assessment in the dedicated study center between 2011 and 2016.

### Standard Protocol Approvals, Registrations, and Participant Consents

The Rotterdam Study was approved by the medical ethics committee of the Erasmus MC (registration MEC 02.1015) and by the Dutch Ministry of Health, Welfare and Sport (Population Screening Act WBO, license 1071272-159521-PG). The Rotterdam Study was entered into the Netherlands National Trial Register and the World Health Organization’s International Clinical Trials Registry Platform under a shared catalog number (NTR6831). All participants provided written informed consent to participate in the study and to have their information obtained from treating physicians.

### Data Availability

Requests for data from the Rotterdam Study should be directed toward the management team of the study (secretariat.epi@erasmusmc.nl), which has a protocol for approving data requests. Because of restrictions based on privacy regulations and the informed consent of the participants, data cannot be made freely available in a public repository.

### Tinnitus Assessment

Tinnitus assessment was performed through a home interview. Participants were asked if they experienced sounds in the head or one of the ears (eg, whizzing, peeping, or humming) without an objective external sound source being present. Possible answers to this question were as follows: *No, never; Yes, less than once a week; Yes, more than once a week but not daily*; and *Yes, daily*.

For the current study, tinnitus was investigated as a binary variable: either not present (*No, never; Yes, less than once a week*) or present (*Yes, more than once a week but not daily; Yes, daily*). Because of the heterogeneity of the origin and the often temporary character of tinnitus, the presence of less than once a week was not recorded as prevalent tinnitus. All participants who answered that they experienced tinnitus were asked whether it interferes with daily life (*Yes* or *No*).

Only participants experiencing tinnitus on a daily basis were asked to fill out the THI-s.^24^ This inventory consists of 10 items, with a possible score of 0, 2, or 4 per item, which includes questions on the interference of tinnitus in daily life. A score ≥16 represented a moderate/severe handicap.^24,25^

### Hearing Assessment

Audiometric assessment was performed by 1 trained health care professional in a soundproof booth. For the audiometric assessment, a computer-based audiometry system (version 210.2.6 with AudioNigma interface; Decos Technology Group) and TDH-39 headphones were used.^23^ To determine hearing levels in decibel hearing level (dB HL), pure tone audiometry was used according to ISO standard 8253-1.^26^ Air conduction thresholds for both ears were measured on different frequencies (0.25, 0.5, 1, 2, 4, and 8 kHz). Masking was performed according to the method of Hood.^27^ Conductive hearing losses (air-bone gap >15 dB HL) were not excluded, as the origin of the hearing loss does not seem to matter in tinnitus induction.^28^ The worse-hearing ear was determined by taking the average decibel hearing level over all measured frequencies. The worse-hearing ear is chosen as this is the most probable ear for tinnitus to occur in.^29^ Pure tone average hearing thresholds, averaged over 0.5, 1, 2, and 4 kHz, were determined according to the worse-hearing ear.^29^ Hearing impairment was determined as an average threshold ≥25 dB HL.^30^

### Covariables

Sex, age (years), and highest achieved educational level were investigated as covariables. Educational level was categorized as lower, middle, or higher education according to the UNESCO International Standard Classification of Education.^31^

### Statistical Analysis

We investigated the prevalence of tinnitus in several steps. First, we compared the differences in demographic characteristics (sex, age, and highest achieved education) between participants with and without tinnitus. We used a *t* test, 1-way analysis of variance, Mann-Whitney *U* test, and χ^2^ test when appropriate. Second, we performed a multivariable logistic regression analysis for the association between hearing impairment and tinnitus, adjusted for sex and age. We repeated this analysis while stratifying in 5-year age groups. Next, we described the severity of the tinnitus complaints, as reported with the THI-s. The THI-s score was described as median (interquartile range) and as percentage with a score ≥16 (ie, reporting a relevant tinnitus-associated handicap). We compared demographics between participants with a relevant handicap and a low handicap. Finally, we performed a sensitivity analysis with an altered definition of tinnitus (only daily) and no tinnitus (never tinnitus) in the demographics of the population and between participants with and without tinnitus according to this definition.

## Results

### Tinnitus Prevalence and Demographic Characteristics

**Figure 1.**
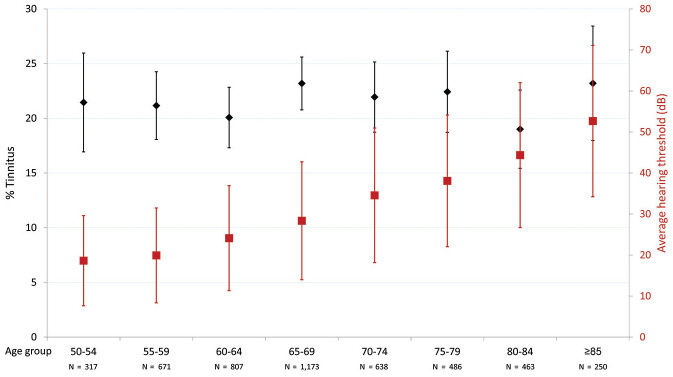
In participants with hearing assessment (n = 4805), tinnitus prevalence (95% CI) and average hearing threshold (±1 SD) per 5-year age groups. Hearing threshold: 0.5, 1, 2, and 4 kHz in the worse-hearing ear.

We found that 21.4% of 6098 participants reported tinnitus (**Table 1**). The prevalence of tinnitus did not vary significantly among the age groups: it ranged between 23.2% in the 65- to 69-year-old group and 19.9% in the 80- to 84-year-old group (χ^2^ test, *P* = .585; **Figure 1**). Participants with prevalent tinnitus were more often male than participants without tinnitus (46.5% vs 41.0%, *P* < .001). A similar difference in the proportion of males was found in the age groups spanning 60 to 74 years, not in the other age groups. There was no difference in highest achieved education between the participants with and without tinnitus, neither in the entire population nor by age group.

**Table 1.** Participant Characteristics Comparing Tinnitus With No Tinnitus (N = 6098).

	Population, No. (%)			
	Total	Tinnitus	No tinnitus	*P* value
Participants	6098	1304 (21.4)	4794 (78.6)	
Male	2570 (42.1)	606 (46.5)	1964 (41.0)	<.001
Age, y	69.4 (10.1)	69.3 (9.8)	69.5 (10.2)	.644
Age group				.585
50-54	382 (6.3)	79 (6.1)	303 (6.3)	
55-59	829 (13.6)	172 (13.2)	657 (13.7)	
60-64	953 (15.6)	191 (14.6)	762 (15.9)	
65-69	1382 (22.7)	321 (24.6)	1061 (22.1)	
70-74	780 (12.8)	174 (13.3)	606 (12.6)	
75-79	622 (10.2)	135 (10.4)	487 (10.2)	
80-84	677 (11.1)	135 (10.4)	542 (11.3)	
≥85	473 (7.8)	97 (7.4)	376 (7.8)	
Education				.149
Lower	2953 (48.4)	661 (50.7)	2292 (47.8)	
Middle	1749 (29.4)	374 (28.7)	1420 (29.6)	
Higher	1351 (22.2)	269 (20.6)	1082 (22.6)	
Hearing (n = 4805)				
Threshold, dB HL^a^	30.5 (17.3)	35.4 (19.2)	29.1 (16.5)	<.001
Impairment ≥25 dB HL^b^	1547 (32.2)	449 (43.2)	1098 (29.2)	<.001
Tinnitus impairment, daily life	160 (2.6)	160 (12.3)	—	
THI-s (n = 663)				
Score^c^	4 (0, 10)	4 (0, 10)	—	
≥16^d^	97 (1.6)	97 (14.6)	—	

Abbreviations: dB HL, decibel hearing level; THI-s, Tinnitus Handicap Inventory–screening version.

a Mean (SD) for normally distributed continuous variable.

b Hearing impairment was averaged over the 0.5-, 1-, 2-, 4-kHz frequencies in the worst ear.

c Median (interquartile range) for nonnormally distributed continuous variable.

d Participants experiencing a moderate/severe handicap from tinnitus.

### Tinnitus and Hearing Impairment

The average hearing threshold in the study population was 30.5 dB HL (SD, 17.3; **Table 1**). In all age groups, except ≥85 years, participants with tinnitus had a significantly higher average hearing threshold (**Table 2**).

**Table 2.** Participant Characteristics Comparing Tinnitus With No Tinnitus per 5-Year Age Category.

		Population, No. (%)			
	No.	Total	Tinnitus	No tinnitus	*P* value
50-54 y					
Participants	382	382	79 (20.7)	303 (79.3)	
Male	382	159 (41.6)	36 (45.6)	123 (40.6)	.424
Education	382				.457
Lower		125 (32.7)	28 (35.4)	97 (32.0)	
Middle		144 (37.7)	25 (31.6)	119 (39.3)	
Higher		113 (29.6)	26 (32.9)	87 (28.7)	
Hearing					
Threshold, dB HL^a^	317	18.7 (11.0)	22.1 (10.8)	17.7 (10.9)	.004
Impairment ≥25 dB HL^b^	317	21 (6.6)	11 (16.2)	10 (4.0)	<.001
Tinnitus impairment daily life	382	8 (2.1)	8 (10.1)	—	
THI-s					
Score^c^	39	4 (0, 8)	4 (0, 8)	—	
≥16^d^	39	6 (1.6)	6 (15.4)	—	
55-59 y					
Participants	829	829	172 (20.7)	657 (79.3)	
Male	829	360 (43.4)	80 (46.5)	280 (42.6)	.406
Education	829				.711
Lower		289 (34.9)	63 (36.6)	226 (34.4)	
Middle		263 (31.7)	56 (32.6)	207 (31.5)	
Higher		277 (33.4)	53 (30.8)	224 (34.1)	
Hearing					
Threshold, dB HL^a^	671	19.9 (11.6)	23.4 (14.8)	19.0 (10.3)	.001
Impairment ≥25 dB HL^b^	671	56 (8.3)	18 (12.7)	38 (7.2)	.036
Tinnitus impairment daily life	829	27 (3.3)	27 (15.9)	—	
THI-s					
Score^c^	87	4 (0, 12)	4 (0, 12)	—	
≥16^d^	87	14 (1.7)	14 (16.1)	—	
60-64 y					
Participants	953	953	191 (20.0)	762 (80.0)	
Male	953	406 (42.6)	98 (51.3)	308 (40.4)	.007
Education	953				.596
Lower		439 (46.1)	93 (48.7)	346 (45.4)	
Middle		253 (26.5)	51 (26.7)	202 (26.5)	
Higher		261 (27.4)	47 (24.6)	214 (28.1)	
Hearing					
Threshold, dB HL^a^	807	24.1 (12.8)	28.6 (13.2)	23.0 (12.4)	<.001
Impairment ≥25 dB HL^b^	807	117 (14.5)	38 (23.5)	79 (12.2)	<.001
Tinnitus impairment daily life	953	27 (2.8)	27 (14.1)	—	
THI-s					
Score^c^	90	6 (0, 14)	6 (0, 14)	—	
≥16^d^	90	22 (2.3)	22 (24.4)	—	
65-69 y					
Participants	1382	1,382	321 (23.2)	1,061 (76.8)	
Male	1382	624 (45.2)	167 (52.0)	457 (43.1)	.005
Education	1382				.192
Lower		691 (50.0)	154 (48.0)	537 (50.6)	
Middle		356 (25.8)	95 (29.6)	261 (24.6)	
Higher		335 (24.2)	72 (22.4)	263 (24.8)	
Hearing					
Threshold, dB HL^a^	1173	28.4 (14.4)	34.0 (17.3)	26.7 (12.9)	<.001
Impairment ≥25 dB HL^b^	1173	278 (23.7)	105 (38.6)	173 (19.2)	<.001
Tinnitus impairment daily life	1382	37 (2.7)	37 (11.5)	—	
THI-s					
Score^c^	165	4 (0, 10)	4 (0, 10)	—	
≥16^d^	165	21 (1.5)	21 (12.7)	—	
70-74 y					
Participants	780	780	174 (22.3)	606 (77.7)	
Male	780	340 (43.6)	92 (52.9)	248 (40.9)	.005
Education	780				.384
Lower		419 (53.7)	100 (57.5)	319 (52.6)	
Middle		229 (29.4)	50 (28.7)	179 (29.5)	
Higher		132 (16.9)	24 (13.8)	108 (17.8)	
Hearing					
Threshold, dB HL^a^	638	34.6 (16.4)	40.5 (20.3)	32.9 (14.7)	<.001
Impairment ≥25 dB HL^b^	638	258 (40.4)	74 (52.9)	184 (36.9)	.001
Tinnitus impairment daily life	780	20 (2.6)	20 (11.5)	—	
THI-s					
Score^c^	75	2 (0, 8)	2 (0, 8)	—	
≥16^d^	75	10 (1.3)	10 (13.3)	—	
75-79 y					
Participants	622	622	135 (21.7)	487 (78.3)	
Male	622	260 (41.8)	57 (42.2)	203 (41.7)	.911
Education	622				.030
Lower		337 (54.2)	84 (62.2)	253 (52.0)	
Middle		191 (30.7)	29 (21.5)	162 (33.3)	
Higher		94 (15.1)	22 (16.3)	72 (14.8)	
Hearing					
Threshold, dB HL^a^	486	38.1 (16.0)	44.5 (17.2)	36.2 (15.2)	<.001
Impairment ≥25 dB HL^b^	486	274 (56.4)	78 (71.6)	196 (52.0)	<.001
Tinnitus impairment daily life	622	17 (2.7)	17 (12.5)	—	
THI-s					
Score^c^	79	4 (0, 10)	4 (0, 10)	—	
≥16^d^	79	11 (1.7)	11 (13.9)	—	
80-84 y					
Participants	677	677	135 (19.9)	542 (80.1)	
Male	677	276 (40.8)	48 (35.6)	228 (42.1)	.168
Education	677				.366
Lower		351 (51.8)	77 (57.0)	274 (50.6)	
Middle		228 (33.7)	42 (31.1)	186 (34.3)	
Higher		98 (14.5)	16 (11.9)	82 (15.1)	
Hearing					
Threshold, dB HL^a^	463	44.4 (17.7)	49.5 (19.1)	43.2 (17.1)	.003
Impairment ≥25 dB HL^b^	463	325 (70.2)	71 (80.7)	254 (67.7)	.017
Tinnitus impairment daily life	677	16 (2.4)	16 (11.9)	—	
THI-s					
Score^c^	82	4 (0, 8)	4 (0, 8)	—	
≥16^d^	82	9 (1.3)	9 (11.0)	—	
≥85 y					
Participants	473	473	97 (20.5)	376 (79.5)	
Male	473	145 (30.7)	28 (28.9)	117 (31.1)	.668
Education	473				.964
Lower		302 (63.8)	62 (63.9)	240 (63.8)	
Middle		130 (27.5)	26 (26.8)	104 (27.7)	
Higher		41 (8.7)	9 (9.3)	32 (8.5)	
Hearing					
Threshold, dB HL^a^	250	52.7 (18.4)	55.3 (19.2)	51.9 (18.2)	.221
Impairment ≥25 dB HL^b^	250	218 (87.2)	54 (93.1)	164 (85.4)	.125
Tinnitus impairment daily life	473	8 (1.7)	8 (8.2)	—	
THI-s					
Score^c^	46	2 (0, 4)	2 (0, 4)	—	
≥16^d^	46	4 (0.8)	4 (8.7)	—	

Abbreviations: dB HL, decibel hearing level; THI-s, Tinnitus Handicap Inventory–screening version.

a Mean (SD) for normally distributed continuous variable.

b Hearing impairment was averaged over the 0.5-, 1-, 2-, 4-kHz frequencies in the worst ear.

c Median (interquartile range) for nonnormally distributed continuous variable.

d Participants experiencing a moderate/severe handicap from tinnitus.

**Figure 2.**
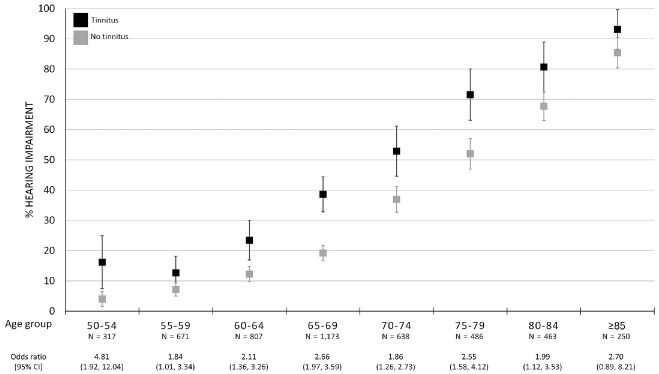
The prevalence of hearing impairment in participants with and without tinnitus (n = 4805), per age category. Odds ratios are adjusted for age and sex. Hearing impairment: ≥25 dB HL over 0.5 to 4 kHz in worse-hearing ear. Values represent proportion ± 95% CI.

The prevalence of hearing impairment was 25.4% in the entire population and significantly higher in the participants with tinnitus as compared with those without (43.2% vs 29.2%, *P* < .0001; **Table 1**). Participants with prevalent tinnitus in the youngest age group in our population (50-54 years) more often had hearing impairment (16.2%) than participants without tinnitus (4.0%, *P* < .001; **Table 2**, **Figure 2**). The increase of the prevalence of hearing impairment is similar in participants with and without tinnitus. Participants with hearing impairment were twice as likely to have tinnitus as compared with participants without hearing impairment (odds ratio, 2.27; 95% CI 1.92-2.69), a result that was found across all age groups.

### Tinnitus Handicap

Of all participants with tinnitus (n = 1304), 160 (12.3%) reported that their tinnitus interfered with daily life. This reflected 2.6% of the entire population. The THI-s was available for 76% of the participants with daily tinnitus (**Table 1**). The median THI-s score was 4 (interquartile range, 0-10), representing a slight handicap or none. A relevant tinnitus handicap (score ≥16) was found in 14.6% (n = 97) of the participants who filled out the THI-s. The median THI-s score was 4 for almost all age categories. The prevalence of a relevant handicap hardly showed differences among age categories, except for a slightly higher percentage in the group 60 to 64 years old (**Table 2**). We also did not find a significant difference between the sexes (male, 12.2%; female, 16.8%; *P* = .092) or by hearing threshold (no hearing impairment, 14.0%; hearing impairment, 16.1%; *P* = .481).

### Sensitivity Analysis

Finally, we ran a sensitivity analysis in which *tinnitus* was defined as daily tinnitus and *no tinnitus* as never tinnitus. Here, we found that an altered definition of tinnitus did not lead to significant differences in the results (**Table 3** vs **Table 1**).

**Table 3.** Sensitivity Analysis Comparing Daily Tinnitus With Never Tinnitus.

		Population, No. (%)			
	No.	Total	Tinnitus	No tinnitus	*P* value
Participants	4920	4920	827 (16.8)	4093 (83.2)	
Male	4920	2100 (42.7)	399 (48.2)	1701 (41.6)	<.001
Age, y	4920	69.6 (10.1)	69.5 (9.6)	69.7 (10.2)	.690
Age group	4920				.170
50-54		290 (5.8)	45 (5.4)	245 (6.0)	
55-59		670 (13.6)	104 (12.5)	566 (13.8)	
60-64		751 (15.3)	115 (13.9)	636 (15.5)	
65-69		1105 (22.5)	210 (25.4)	895 (21.9)	
70-74		646 (13.1)	119 (14.4)	527 (12.9)	
75-79		506 (10.3)	89 (10.8)	417 (10.2)	
80-84		572 (11.6)	93 (11.2)	479 (11.7)	
≥85		380 (7.7)	52 (6.3)	328 (8.0)	
Education	4920				.478
Lower		2357 (47.9)	409 (49.5)	1948 (47.6)	
Middle		1467 (29.8)	246 (29.7)	1221 (29.8)	
Higher		1096 (22.3)	172 (20.8)	924 (22.6)	
Hearing					
Threshold, dB HL^a^	3896	30.4 (17.1)	37.0 (19.2)	29.0 (16.3)	<.001
Impairment ≥25 dB HL^b^	3896	1264 (25.7)	320 (47.5)	944 (29.3)	<.001
Tinnitus impairment daily life	4920	110 (2.2)	110 (13.3)	—	
THI-s					
Score^c^	663	4 (0, 10)	4 (0, 10)	—	
≥16^d^	663	97 (2.0)	97 (11.7)	—	

Abbreviations: dB HL, decibel hearing level; THI-s, Tinnitus Handicap Inventory–screening version.

a Mean (SD) for normally distributed continuous variable.

b Hearing impairment was averaged over the 0.5-, 1-, 2-, 4-kHz frequencies in the worst ear.

c Median (interquartile range) for nonnormally distributed continuous variable.

d Participants experiencing a moderate/severe handicap from tinnitus.

## Discussion

In this study, we found that the prevalence of tinnitus was 21.4% in a general Dutch population-based sample of older adults (≥50 years), using a definition of tinnitus being present more than once a week regardless of the tinnitus burden. For 1 out of 10 persons with tinnitus, the presence of tinnitus interfered with their daily life. Furthermore, participants with hearing impairment were twice as likely to have tinnitus. Despite the age-dependent occurrence of hearing impairment, no such age dependency was found for tinnitus.

In this study, we found a similar prevalence of tinnitus over the age groups, whereas the proportion of participants with hearing impairment was, as expected, much higher in the older groups. Interestingly, we found a similar increase of percentage hearing impairment above the age of 54 years for the tinnitus and no-tinnitus groups. This suggests that tinnitus in itself is, unlike hearing impairment, probably not associated with aging processes. We propose several possible mechanisms for this. First, aging does not put individuals at greater risk of developing tinnitus. This implies that age-related change/decline of the brain does not lead to an increased vulnerability for developing tinnitus. Second, although hearing loss in general is an important risk factor for tinnitus, age-related aspects of hearing impairment are not likely to induce tinnitus. One of the explanations may be the gradual development of age-related hearing impairment. It is suggested that a sudden lack of input to the brain from the cochlea can result in tinnitus.^32-35^ In contrast to this hypothesis stands age-related hearing impairment, which is a slowly progressing disease of the auditory system; therefore, the brain has time to adjust to the increasing lack of input.^35^ Another possible explanation is that the pathophysiology of age-related hearing impairment is principally different from other types of hearing loss that are more likely to induce tinnitus (eg, noise-induced hearing loss).^33,36,37^ We therefore hypothesize that tinnitus and hearing impairment in the elderly co-occur, but the age-related aspect of hearing impairment does not seem to contribute to the found association between hearing impairment and tinnitus.

The reported prevalence of tinnitus in our cohort, 21.5%, is in the middle of the range reported by the McCormack et al review (5.1%-42.7%), consisting of larger and smaller populations.^1^ Specifically, tinnitus prevalence from other large population-based studies ranges from 9.6% up to 30.3%.^14,15,22,38-40^ To our knowledge, no other study has yet reported the prevalence of tinnitus in 5-year age intervals, in which we unexpectedly found no differences. This is in contrast to what McCormack et al reported in their review: “The prevalence figures generally show an increase in tinnitus prevalence as age increases.”^1^ It should be noted, though, that this statement is based on studies reporting tinnitus prevalence in populations aged ≥20 years and not solely in an elderly population, as in our study. Only a few studies describe prevalence trends in 10-year intervals in populations consisting of older participants (>45 years). These studies report ambiguous conclusions about tinnitus prevalence in these older participants: both an increased prevalence^11,14^ and a similar prevalence^12,13^ with increasing age. Interestingly, these 4 studies are comparable in their assessment of tinnitus and consist of larger populations (N > 1320), similar to our current study.

Comparing tinnitus prevalence among studies is complicated, as there is no gold standard for the assessment. The frequency of tinnitus being present is one of the main differences in definition among studies. This frequency ranges between *Daily >5 minutes* and *Ever*.^1^ For example, when we alter the definition of tinnitus in our study, the reported prevalence changes as well. The prevalence increases from 21.4% to 32.9% when a broader definition is applied, including any form of tinnitus. This might result in effect dilution, as it increases the chance of misclassifying temporary tinnitus related to specific conditions (eg, noise exposure) as chronic tinnitus. Conversely, if we classify participants with daily tinnitus as having prevalent tinnitus, the prevalence in our population decreases toward 13.6%. This number decreases further toward 2.1% in our population when tinnitus is defined as experiencing it on a daily basis and when it interferes with daily life.

Population-based studies have shown that the handicap associated with tinnitus is generally mild, yet for some, it interferes with life on a daily basis.^4,41,42^ This is similar to what we found in the current study: bothersome tinnitus was reported by 1 of 10 participants with prevalent tinnitus. Of these participants, most answered being bothered by tinnitus on a daily basis. Of the participants with daily tinnitus, 11.7% had a score >16 on the THI-s, reflecting moderate or worse handicap associated with tinnitus. One should be careful to extrapolate these results to clinical tinnitus populations. The clinical tinnitus population is a highly selected group with a large burden of disease, which is probably only a subgroup of our participants who report tinnitus to interfere with daily life.^10^

Even though hearing impairment is regarded as the main risk factor for tinnitus, there are other potential risk factors for tinnitus that may affect the prevalence, such as depression, anxiety, cardiovascular risks, or genetics.^4,15,43-45^ Deteriorated mental health is often reported in clinical tinnitus populations and to be associated with a high tinnitus burden.^44-46^ As the Rotterdam Study consists of relatively healthy older individuals with a low tinnitus burden, we do not expect this to affect the overall tinnitus prevalence reported in the present study.

The current study is one of the larger population-based studies investigating tinnitus prevalence and its relation to hearing impairment measured with pure tone audiometry. The large sample size and pure tone audiometry allowed for proper investigation of the association of hearing impairment in an elderly population. Some limitations in the current study should also be acknowledged. First, it remains unknown in which ear the tinnitus is present, which would have allowed for closer investigation of the association with hearing impairment. Second, no information was available on tinnitus onset and duration. Third, this study was of a cross-sectional origin, limiting the ability to infer on causality.

To conclude, tinnitus is present in 1 out of 5 older adults, and every 1 out of 10 with tinnitus experience severe tinnitus that is interfering with daily life. Participants with hearing impairment were twice as likely to have tinnitus as compared with participants without hearing impairment. In spite of the strong age-related character of hearing impairment, no such age dependency was found for the prevalence of tinnitus.

## References

[1] McCormack A Edmondson-Jones M Somerset S, et al. A systematic review of the reporting of tinnitus prevalence and severity. Hear Res. 2016;337:70-79. 27246985 10.1016/j.heares.2016.05.009

[2] Cima RF Vlaeyen JW Maes IH, et al. Tinnitus interferes with daily life activities: a psychometric examination of the Tinnitus Disability Index. Ear Hear. 2011;32:623-633. 21336139 10.1097/AUD.0b013e31820dd411

[3] Friberg E Jansson C Mittendorfer-Rutz E, et al. Sickness absence due to otoaudiological diagnoses and risk of disability pension: a nationwide Swedish prospective cohort study. PLoS One. 2012;7:e29966. 10.1371/journal.pone.0029966 PMC3257229 22253839

[4] Kim HJ Lee HJ An SY, et al. Analysis of the prevalence and associated risk factors of tinnitus in adults. PLoS One. 2015;10:e0127578. 10.1371/journal.pone.0127578 PMC4447366 26020239

[5] Hannaford PC Simpson JA Bisset AF, et al. The prevalence of ear, nose and throat problems in the community: results from a national cross-sectional postal survey in Scotland. Fam Pract. 2005;22:227-233. 15772117 10.1093/fampra/cmi004

[6] Lee DY Kim YH. Relationship between diet and tinnitus: Korea National Health and Nutrition Examination Survey. Clin Exp Otorhinolaryngol. 2018;11:158-165. 29433160 10.21053/ceo.2017.01221 PMC6102331

[7] Kontis V Bennett JE Mathers CD, et al. Future life expectancy in 35 industrialised countries: projections with a Bayesian model ensemble. Lancet. 2017;389:1323-1335. 28236464 10.1016/S0140-6736(16)32381-9 PMC5387671

[8] GBD 2016 Disease and Injury Incidence and Prevalence Collaborators. Global, regional, and national incidence, prevalence, and years lived with disability for 328 diseases and injuries for 195 countries, 1990-2016: a systematic analysis for the Global Burden of Disease Study 2016. Lancet. 2017;390:1211-1259. 28919117 10.1016/S0140-6736(17)32154-2 PMC5605509

[9] Stohler NA Reinau D Jick SS, et al. A study on the epidemiology of tinnitus in the United Kingdom. Clin Epidemiol. 2019;11:855-871. 31572016 10.2147/CLEP.S213136 PMC6750864

[10] Martinez C Wallenhorst C McFerran D, et al. Incidence rates of clinically significant tinnitus: 10-year trend from a cohort study in England. Ear Hear. 2015;36:e69-e75. 10.1097/AUD.0000000000000121 PMC4415963 25470370

[11] Fujii K Nagata C Nakamura K, et al. Prevalence of tinnitus in community-dwelling Japanese adults. J Epidemiol. 2011;21:299-304. 21646745 10.2188/jea.JE20100124 PMC3899423

[12] Michikawa T Nishiwaki Y Kikuchi Y, et al. Prevalence and factors associated with tinnitus: a community-based study of Japanese elders. J Epidemiol. 2010;20:271-276. 20501961 10.2188/jea.JE20090121 PMC3900786

[13] Nondahl DM Cruickshanks KJ Wiley TL, et al. Prevalence and 5-year incidence of tinnitus among older adults: the epidemiology of hearing loss study. J Am Acad Audiol. 2002;13:323-331. 12141389

[14] Sindhusake D Mitchell P Newall P, et al. Prevalence and characteristics of tinnitus in older adults: the Blue Mountains Hearing Study. Int J Audiol. 2003;42:289-294. 12916702 10.3109/14992020309078348

[15] Shargorodsky J Curhan GC Farwell WR. Prevalence and characteristics of tinnitus among US adults. Am J Med. 2010;123:711-718. 20670725 10.1016/j.amjmed.2010.02.015

[16] Dawes P Fortnum H Moore DR, et al. Hearing in middle age: a population snapshot of 40- to 69-year olds in the United Kingdom. Ear Hear. 2014;35:e44-e51. 10.1097/AUD.0000000000000010 PMC4264521 24518430

[17] Demeester K van Wieringen A Hendrickx JJ, et al. Prevalence of tinnitus and audiometric shape. B-ENT. 2007;3(suppl 7):37-49. 18225607

[18] Hannula S Bloigu R Majamaa K, et al. Self-reported hearing problems among older adults: prevalence and comparison to measured hearing impairment. J Am Acad Audiol. 2011;22:550-559. 22031679 10.3766/jaaa.22.8.7

[19] Lasisi AO Abdullahi M. The inner ear in patients with nasal allergy. J Natl Med Assoc. 2008;100:903-905. 18717140 10.1016/s0027-9684(15)31403-6

[20] Parving A Hein HO Suadicani P, et al. Epidemiology of hearing disorders: some factors affecting hearing. The Copenhagen Male Study. Scand Audiol. 1993;22:101-107. 8321994 10.3109/01050399309046025

[21] McCormack A Edmondson-Jones M Fortnum H, et al. The prevalence of tinnitus and the relationship with neuroticism in a middle-aged UK population. J Psychosom Res. 2014;76:56-60. 24360142 10.1016/j.jpsychores.2013.08.018

[22] Nondahl DM Cruickshanks KJ Dalton DS, et al. The impact of tinnitus on quality of life in older adults. J Am Acad Audiol. 2007;18:257-266. 17479618 10.3766/jaaa.18.3.7

[23] Ikram MA Brusselle G Ghanbari M, et al. Objectives, design and main findings until 2020 from the Rotterdam Study. Eur J Epidemiol. 2020;35(5):483-517. 32367290 10.1007/s10654-020-00640-5 PMC7250962

[24] Newman CW Sandridge SA Bolek L. Development and psychometric adequacy of the screening version of the Tinnitus Handicap Inventory. Otol Neurotol. 2008;29:276-281. 18277308 10.1097/MAO.0b013e31816569c4

[25] Newman CW Sandridge SA Jacobson GP. Psychometric adequacy of the Tinnitus Handicap Inventory (THI) for evaluating treatment outcome. J Am Acad Audiol. 1998;9:153-160. 9564679

[26] ISO. ISO 8253-1:2010. Acoustics—Audiometric Test Methods—Part 1: Pure-Tone Air and Bone Conduction Audiometry. ISO; 2010.

[27] Hood JD. The principles and practice of bone conduction audiometry: a review of the present position. Laryngoscope. 1960;70:1211-1228. 13715685 10.1288/00005537-196009000-00001

[28] Schaette R Turtle C Munro KJ. Reversible induction of phantom auditory sensations through simulated unilateral hearing loss. PLoS One. 2012;7:e35238. 10.1371/journal.pone.0035238 PMC3366980 22675466

[29] Lee HY Kim SJ Chang DS, et al. Tinnitus in the side with better hearing. Am J Otolaryngol. 2019;40:400-403. 30799211 10.1016/j.amjoto.2019.02.009

[30] World Health Organisation. Grades of hearing impairment. Accessed August 2020. http://www.who.int/pbd/deafness/hearing_impairment_grades/en/

[31] UNESCO. International Standard Classification of Education (ISCED). UNESCO; 1976.

[32] Barbee CM James JA Park JH, et al. Effectiveness of auditory measures for detecting hidden hearing loss and/or cochlear synaptopathy: a systematic review. Semin Hear. 2018;39:172-209. 29915454 10.1055/s-0038-1641743 PMC6003814

[33] Haider HF Bojic T Ribeiro SF, et al. Pathophysiology of subjective tinnitus: triggers and maintenance. Front Neurosci. 2018;12:866. 30538616 10.3389/fnins.2018.00866 PMC6277522

[34] Kaltenbach JA. Tinnitus: models and mechanisms. Hear Res. 2011;276:52-60. 21146597 10.1016/j.heares.2010.12.003 PMC3109239

[35] Sedley W. Tinnitus: does gain explain? Neuroscience. 2019;407:213-228. 30690137 10.1016/j.neuroscience.2019.01.027

[36] Langguth B Landgrebe M Schlee W, et al. Different patterns of hearing loss among tinnitus patients: a latent class analysis of a large sample. Front Neurol. 2017;8:46. 28265258 10.3389/fneur.2017.00046 PMC5316929

[37] Pan T Tyler RS Ji H, et al. The relationship between tinnitus pitch and the audiogram. Int J Audiol. 2009;48:277-294. 19842803 10.1080/14992020802581974

[38] Bhatt JM Lin HW Bhattacharyya N. Prevalence, severity, exposures, and treatment patterns of tinnitus in the United States. JAMA Otolaryngol Head Neck Surg. 2016;142:959-965. 27441392 10.1001/jamaoto.2016.1700 PMC5812683

[39] House L Bishop CE Spankovich C, et al. Tinnitus and its risk factors in african americans: the Jackson Heart Study. Laryngoscope. 2018;128:1668-1675. 29193110 10.1002/lary.26964 PMC5975087

[40] Spankovich C Gonzalez VB Su D, et al. Self reported hearing difficulty, tinnitus, and normal audiometric thresholds, the National Health and Nutrition Examination Survey 1999-2002. Hear Res. 2018;358:30-36. 29254853 10.1016/j.heares.2017.12.001

[41] Gopinath B McMahon CM Rochtchina E, et al. Incidence, persistence, and progression of tinnitus symptoms in older adults: the Blue Mountains Hearing Study. Ear Hear. 2010;31:407-412. 20124901 10.1097/AUD.0b013e3181cdb2a2

[42] Henry JA Griest S Zaugg TL, et al. Tinnitus and hearing survey: a screening tool to differentiate bothersome tinnitus from hearing difficulties. Am J Audiol. 2015;24:66-77. 25551458 10.1044/2014_AJA-14-0042 PMC4689225

[43] Vona B. Heritability and tinnitus. JAMA Otolaryngol Head Neck Surg. 2019;145(3):229-230. 30653225 10.1001/jamaoto.2018.3946

[44] Pattyn T Van Den Eede F Vanneste S, et al. Tinnitus and anxiety disorders: a review. Hear Res. 2016;333:255-265. 26342399 10.1016/j.heares.2015.08.014

[45] Salazar JW Meisel K Smith ER, et al. Depression in patients with tinnitus: a systematic review. Otolaryngol Head Neck Surg. 2019;161:28-35. 30909841 10.1177/0194599819835178 PMC7721477

[46] Trevis KJ McLachlan NM Wilson SJ. A systematic review and meta-analysis of psychological functioning in chronic tinnitus. Clin Psychol Rev. 2018;60:62-86. 29366511 10.1016/j.cpr.2017.12.006

